# Choice of library size normalization and statistical methods for differential gene expression analysis in balanced two-group comparisons for RNA-seq studies

**DOI:** 10.1186/s12864-020-6502-7

**Published:** 2020-01-28

**Authors:** Xiaohong Li, Nigel G. F. Cooper, Timothy E. O’Toole, Eric C. Rouchka

**Affiliations:** 1 Department of Anatomical Sciences and Neurobiology, University of Louisville, Louisville, KY USA; 2 Envirome Institute, University of Louisville, Louisville, KY USA; 3 Department of Computer Science and Engineering, University of Louisville, Louisville, KY USA

**Keywords:** RNA-seq, Sample sizes, Normalization, Statistical test, Differentially expressed genes

## Abstract

**Background:**

High-throughput RNA sequencing (RNA-seq) has evolved as an important analytical tool in molecular biology. Although the utility and importance of this technique have grown, uncertainties regarding the proper analysis of RNA-seq data remain. Of primary concern, there is no consensus regarding which normalization and statistical methods are the most appropriate for analyzing this data. The lack of standardized analytical methods leads to uncertainties in data interpretation and study reproducibility, especially with studies reporting high false discovery rates. In this study, we compared a recently developed normalization method, UQ-pgQ2, with three of the most frequently used alternatives including RLE (relative log estimate), TMM (Trimmed-mean M values) and UQ (upper quartile normalization) in the analysis of RNA-seq data. We evaluated the performance of these methods for gene-level differential expression analysis by considering the factors, including: 1) normalization combined with the choice of a Wald test from *DESeq2* and an exact test/QL (Quasi-likelihood) F-Test from *edgeR*; 2) sample sizes in two balanced two-group comparisons; and 3) sequencing read depths.

**Results:**

Using the MAQC RNA-seq datasets with small sample replicates, we found that UQ-pgQ2 normalization combined with an exact test can achieve better performance in term of power and specificity in differential gene expression analysis. However, using an intra-group analysis of false positives from real and simulated data, we found that a Wald test performs better than an exact test when the number of sample replicates is large and that a QL F-test performs the best given sample sizes of 5, 10 and 15 for any normalization. The RLE, TMM and UQ methods performed similarly given a desired sample size.

**Conclusion:**

We found the UQ-pgQ2 method combined with an exact test/QL F-test is the best choice in order to control false positives when the sample size is small. When the sample size is large, UQ-pgQ2 with a QL F-test is a better choice for the type I error control in an intra-group analysis. We observed read depths have a minimal impact for differential gene expression analysis based on the simulated data.

## Background

High-through RNA sequencing (RNA-seq) has been increasingly used in the studies of genomics and transcriptomics over the last decade [1, 2]. Unlike cDNA microarray technology, RNA-seq has wide applications for the identification of novel genes or transcripts, mutations, gene editing and differential gene expression [1, 3–7]. Recent clinical studies demonstrated the utility of RNA-seq in identifying complex disease signatures via transcriptome analysis [8, 9]. Despite this utility and importance, optimal methods for analyzing RNA-seq data remain uncertain.

For each sample in an RNA-seq experiment, millions of reads with a desired read length are mapped to a reference genome by alignment tools such as Bowtie2/TopHat2, STAR and HISAT2 [10–14]. The mapped reads for each gene or transcript are subsequently used to quantify its expression abundance. However, the sample read depth typically varies from one sample to another and a direct comparison of gene expression between samples cannot be performed. Thus, normalization and proper test statistics are critical steps in the analysis of RNA-seq data [15].

Normalization of RNA-seq read counts is an essential procedure that corrects for non-biological variation of samples due to library preparation, sequencing read depth, gene length, mapping bias and other technical issues [16–20]. This ensures proper modeling of biological variations to directly compare and accurately detect expression changes between sample groups. Currently, a number of normalization methods are available to correct for technical variations and biases. These include methods to correct for read depth and transcript lengths, most commonly formulated as RPKM (Reads Per Kilobase per Million mapped reads) and FPKM (Fragments Per Kilobase per Million mapped fragments), which have been implemented in *DEGSeq* and *Cufflinks-CuffDiff* [7, 19, 21, 22]. Other global scaling quantile normalization methods consider either a TC (per-sample total counts) [23], UQ (per-sample 75% upper quartile Q3) [17], Med (per-sample Median Q2) [23], or Q (full quantile) implemented in *Aroma.light* [24]. More complex methods based on a size factor imputed include RLE normalization as implemented in *DESeq2/DESeq* and TMM implemented in *edgeR* for correcting read depth bias [16, 25, 26]. Still other methods normalize by the expression of control genes such as RUV for removing unwanted technical variation across samples [17, 27], GC-content [28], or log_2_ transformed read counts implemented in *voom-limma* [24, 29]. In addition to these traditional normalization methods, two abundance estimation normalization methods have been recently developed. One is called RNA-seq by Expectation-Maximization using a directed graph model (*RSEM*) [30] and the other is *Sailfish* which is an alignment-free abundance estimation using k-mers to index and count RNA-seq reads [31]. More recently we developed a method called UQ-pgQ2 (per-gene Q2 normalization following per-sample upper-quartile global scaling at 75 percentile) for correcting library depths and scaling the reads of each gene into the similar levels across conditions [18, 32].

A number of studies have compared these normalization methods and their impact on the downstream analysis for identification of differentially expressed genes (DEGs) (Table 1). Briefly, the earliest comparison studies reported that UQ normalization followed by an exact test/LRT significantly reduced the length bias of DE from RPKM relative to quantitative Real-Time polymerase chain reaction (qRT-PCR) [17] and *baySeq* with UQ normalization had the highest true positive rates with low false positive rates (FPRs). The observed false discovery rate (FDR) from *edgeR*, *DESeq* and *TSPM* methods was higher than the expected rate of 0.05, while *TSPM* performs the worst when sample sizes were as small as two [33]. In contrast, Rapaport et al. reported that no single method was favorable in all comparisons. They observed that *baySeq* with UQ normalization was the least correlated with qRT-PCR, *Cufflinks*-*CuffDiff* had an inflated number of false positive predictions and voom-limma package had comparable performance as *DESeq* and *edgeR* [34]. Moreover, a recent study based on a Spearman correlation analysis between read counts and qRT-PCR for the two abundance estimation methods (*Sailfish* and *RSEM*) revealed that raw counts (RC) or RPKM seemed to be adequate due to inconsistent results from Sailfish and RSEM, suggesting that normalization methods are not necessary for all sequence data [35]. An extensive evaluation performed by Dillest et al. found that an exact test combined with DESeq/TMM normalization was the best for controlling the FDR below 0.05 for high-count genes while RPKM, TC and Q normalization were suggested to be abandoned [23]. Moreover, several studies summarized that *DESeq* was often too conservative, *edgeR, NBPSeq*, and *EBSeq* were too liberal, and voom/vst-limma had good type I error control with low power for small sample sizes [36–39]. These studies concur that *DESeq* is preferred for controlling the number of false positives while *edgeR* with TMM is slightly preferable for controlling false negatives by achieving higher sensitivity.

**Table 1:** Summary of studies comparing normalization methods for the DEG analysis

References	Normalization methods	Software Packages/ pipelines	Replicates per condition (n)	Conclusions
Bullard et al. 2010 [17]	POLR2A, Q, TC, UQ	Genominator	2, 4	POLR2A and UQ with LRT/Exact test significantly reduced the bias of DE relative to qRT-PCR
Kvam et al. 2012 [33]	DESeq, TMM, UQ	DESeq, edgeR, baySeq, TSPM	2, 4, 5	baySeq with UQ normalization performed best with highest sensitivity and low rates of false positives. But all the methods had an inflated true FDR (> 0.1).
Rapaport F. et al. 2013 [34]	DESeq, TMM, UQ, RPKM, FPKM, Q, voom,	Cuffdiff, DESeq, edgeR, baySeq, PoissonSeq, voom-limma	2, 3	No single method emerged as favorable in all comparisons, but baySeq with UQ method was least correlated with qRT-PCR and Cuffdiff had an inflated number of false positive predictions.
Li et al. 2015 [35]	DESeq, Med, Q, RPKM, RC, TMM, UQ, ERPKM	DESeq, edgeR, Cufflinks-CuffDiff, RSEM, Sailfis	2, 4	RC or RPKM seems to be adequate and the results from Sailfish and RSEM with RC or RPKM are inconsistent, resulting a conclusion of that normalization methods are not necessary for all sequence data.
Dilliest et al. 2013 [23]	DESeq, Med, Q, RPKM, TC, TMM, UQ	DESeq, edgeR, Cufflinks-CuffDiff	2, 3	Exact test from DESeq combined with DESeq/TMM normalization performed best in terms of control of FDR below 0.05 for high-count genes; RPKM, TC and Q should be abandoned in DE gene analysis.
Soneson et al. 2013 [36]	DESeq, TMM, UQ, RPKM, FPKM, voom, vst	DESeq, edgeR, EBSeq, baySeq, NBPSeq,NOIseq, SAMseq, ShrinkSeq,TSPM, limma	2, 5, 10, 11	*DESeq* had poor FDR control with 2 samples and good FDR control for larger sample sizes and low TPR.*edgeR* had poor FDR control with high TPR. Voom/vst-limma had good FDR control, but low power for small sample size.
Seyednasroliah et al. 2013 [37]	DESeq, TMM, UQ, RPKM, FPKM, voom	DESeq, edgeR, baySeq, NOIseq, SAMseq, limma, CuffDiff2, EBSeq	2:6, 8,10,12, 16, 20, 24, 28	DESeq and limma were the safe choice and relatively conservative while *edgeR* and EBSeq were too liberal. *DESeq* and *edgeR* were the best tools
Zhang et al. 2014 [38]	DESeq, TMM, FPKM,	DESeq, edgeR, Cufflinks-CuffDiff	1:6, 8, 14, 20	TMM performed best in terms of sensitivity and DESeq was the best for control false positives. Both were not sensitive to the read depth.
Lin et al. 2016 [39]	DESeq, Med, Q, RPKM, TC, TMM, UQ	DESeq, edgeR and SAS	2, 3, 5	DESeq and TMM normalization methods were recommended compared to the other methods.
Tang et al. 2015 [40]	RLE,TMM, UQ, RPKM, FPKM, Q, voom,	DESeq, DESeq2, edgeR, EBSeq, baySeq, SAMseq, PoissonSeq, voom-limma, TCC	1, 3, 6, 9	In multi-group comparison, the proposed pipeline internally using edgeR was recommended for count data with replicates while this pipeline with *DESeq2* was recommended for data without replicates
Germain et al. 2016 [41]	RLE, TMM, voom, TPM	Cufflinks-CuffDiff, DESeq2, edgeR, voom-limma	3, 5	With benchmarked differential expression analysis, in general voom and e*dgeR* showed the most stable performance and be superior to other methods in most assay with replicates of 3 and 5. But voom significantly underperformed in transcript-level simulation and edgeR shown suboptimal results in the SEQC dataset
Maza E 2016 [42]	TMM, RLE, MRN	DESeq2, edgeR	1	The three methods gave the same results for a simple two-condition comparison withourt replicates.
Costa-Silva et al. 2017 [43]	TMM, RLE, UQ, voom	Limma-Voom, NOIseq, DESeq2, SAMSeq, EBSeq, sleuth, baySeq, edgeR	1:8	Limma-voom, NOIseq and *DESeq2* had more consistent results for DEGs identification
Spies et al. 2019 [44]	Vst, Med, RLE, TMM	DyNB, EBSeq-HMM, FunPat, ImpulseDE2, Imms, next maSigPro, nsgp, splineTC, timeSeq, edgeR, DESeq2	2, 3, 5	*DESeq2* and *edgeR* with a pairwise comparison outperformed TC tools for short time course (< 8 time points) due to high false positive rate except ImpulseDE2, but they were less efficient on longer time series than splineTC and maSigPro tools.

Since *DESeq* with an exact test was overly conservative, *DESeq2* with a Wald test was developed for improving the sensitivity/power [25]. Subsequently, a comparison of RLE normalization from *DESeq2* with other existing methods was performed by several studies (Table 1). In one of these studies, a three-group comparison calculated the area under a Receiver Operating Characteristic (ROC) curve and recommended *edgeR* for count data with replicates while *DESeq2* with RLE normalization was recommended for data without replicates [40]. Another study reported that voom and *edgeR* were generally superior to other methods for controlling the FDR with replicates of 3 and 5, but voom significantly underperformed in transcript-level simulation [41]. In contrast, another study reported that TMM, RLE and MRN gave the same results for a two-condition comparison without replicates [42] while *limma-Voom*, *NOIseq* and *DESeq2* had more consistent results for DEG identification [43]. A recent study using RNA-seq time course data found *DESeq2* and *edgeR* with a pairwise comparison outperformed TC tools for short time course (< 8 time points) due to high FPRs, but they performed worse on longer time series than *splineTC* and *maSigPro* tools [44].

Taken together, these studies showed that TMM and/or RLE associated with *edgeR* and *DESeq2* outperformed the others in terms of overall performance on sensitivity and specificity [17, 23, 33, 34, 36, 37, 39–41, 43, 44]. However, these studies also reported that TMM and UQ normalizations were too liberal or oversensitive, resulting in a large number of false positives, while RLE implemented in *DESeq* with an exact test was too conservative [23, 36, 37]. A recent study concluded that RLE/*DESeq2* with a Wald test improves sensitivity compared with a previous version of RLE/*DESeq* with an exact test. But this comes with a trade-off for a relatively higher FPR [25]. Later studies reported that the actual FDR produced from TMM/*edgeR* with an exact test, and RLE/*DESeq2* with a Wald test, was not controlled well in many cases [18, 23, 33, 36, 37]. Most recently, *edgeR* offered a quasi-likelihood (QL) F-test for testing DE genes using negative binomial generalized models, which was considered to be a preferred choice for the uncertainty in estimating the dispersion for each gene when sample sizes were small [45]. In our recent study, we found that UQ-pgQ2 normalization combined with an exact test from *edegR* performed slightly better than TMM and RLE in terms of FDR when using MAQC data and simulated data. However, all the methods had an inflated FDR using MAQC datasets [18]. Thus, it remains unclear which combination of normalization and test statistics can minimize the number of false positives while taking into consideration of sample size and read depth variations. While studies comparing different normalization methods have been widely reported and discussed, this issue for the evaluation of newly developed normalization and testing statistical methods has not been adequately addressed.

In this study, we evaluated the performance of two commonly used packages (*DESeq2* and *edgeR*) with three statistical tests (exact test, QL F-test and Wald test), the three most frequently used normalization methods (RLE, TMM and UQ) and the more recently proposed two-step normalization (UQ-pgQ2). Two benchmark MAQC (Microarray Quality Control Project) datasets [34, 46, 47], five real RNA-seq datasets from The Cancer Genome Atlas (TCGA) website [48], and simulated data with varying read depths are used in this study.

## Results

### Statistical analysis of MAQC2 and MAQC3 for the combined methods

In our previous study, we evaluated the effect of normalization methods including DESeq, TMM, UQ-pgQ2 and UQ based on DEG analysis using two MAQC datasets and an exact test/*edgeR*. In this study, the effects of the Wald test/*DESeq2*, exact test/QL F-test from *edgeR* and t-test/voom-limma were used to evaluate the normalization and test statistical methods.

The number of true positive (TP) and false positive (FP) genes calculated were based on the number of DEGs identified from MAQC RNA-seq data given a nominal FDR cutoff 0.05, and the total number of TPs and true negatives (TNs) were based on qRT-PCR data. We also calculated the positive predictive value (PPV), the actual FDR, sensitivity and specificity for both datasets (Table 2). Using MAQC2 data, the analysis results show that UQ-pgQ2 combined with an exact test/*edgeR* has the highest specificity (85.1%) with the lowest actual FDR (0.055) while the others ranged from 37.8 to 45.3% with a FDR greater than 0.1 and slightly lower sensitivity (96.7%). An exact test*/*TMM has the highest sensitivity (98.5%) while the others ranged from 96.7 to 97.4%. The UQ approach performed the worst in both sensitivity and specificity, consistent with other findings [18].

**Table 2:** Statistical analysis of DEGs from four normalization and test statistics given a nominal FDR ≤ 0.05. Listed are the number of TP and FP genes, the observed FDR and the PPV, sensitivity and specificity using MAQC datasets

Data	Statistical test (package)	Methods	# of TP	# of FP	PPV	Actual FDR	Sensitivity	Specificity
MAQC2 (*n* = 2)	Exact test (*edgeR*)	UQ-pgQ2	377	22	0.945	0.055	0.967	0.851
		TMM	384	81	0.826	0.174	0.985	0.453
		RLE	380	91	0.807	0.193	0.974	0.385
		UQ	379	92	0.805	0.195	0.972	0.378
	Wald test (*DESeq2*)	UQ-pgQ2	385	49	0.887	0.113	0.987	0.669
		UQ	374	80	0.824	0.176	0.959	0.460
		TMM	374	82	0.820	0.180	0.959	0.446
		RLE	376	83	0.819	0.181	0.964	0.439
	T-test (voom-limma)	UQ-pgQ2 & UQ	363	76	0.827	0.173	0.931	0.487
		TMM	373	97	0.794	0.206	0.956	0.345
		RLE	364	92	0.798	0.202	0.933	0.378
	QL F-test (*edgeR*)	UQ-pgQ2	387	59	0.868	0.132	0.997	0.587
		TMM	385	103	0.789	0.211	0.992	0.280
		RLE & UQ	385	108	0.781	0.219	0.992	0.245
MAQC3 (*n* = 5)	Exact test (*edgeR*)	UQ-pgQ2	383	51	0.882	0.118	0.987	0.643
		TMM	386	93	0.806	0.194	0.995	0.350
		RLE	386	98	0.798	0.202	0.995	0.315
		UQ	386	98	0.798	0.202	0.995	0.315
	Wald test (*DESeq2*)	UQ-pgQ2	384	83	0.822	0.178	0.990	0.420
		UQ	387	101	0.793	0.207	0.997	0.294
		TMM	386	103	0.789	0.211	0.995	0.280
		RLE	385	102	0.791	0.209	0.992	0.287
	T-test (voom-limma)	UQ-pgQ2 & UQ	362	58	0.862	0.138	0.932	0.594
		RLE	376	64	0.856	0.146	0.969	0.552
		TMM	350	60	0.853	0.146	0.902	0.580
	QL F-test (*edgeR*)	UQ-pgQ2	382	85	0.818	0.182	0.985	0.406
		TMM	385	99	0.796	0.205	0.992	0.308
		RLE	386	105	0.786	0.214	0.995	0.267
		UQ	386	104	0.788	0.212	0.995	0.273

While using a Wald test*,* the results show that UQ-pgQ2 outperformed the others with the highest specificity (66.9% compared to the others from 43.9 to 46.0%) and a slightly higher sensitivity (98.7% compared to the others from 95.9 to 96.4%). RLE has a slightly higher sensitivity (96.4%) than the TMM and UQ methods while having a tradeoff of lower specificity. When using the recently proposed QL F-test*,* the results show that UQ-pgQ2 has the highest specificity (58.7% compared to the others arranged from 24.5 to 28.0%) and the highest sensitivity (99.7% compared to the others 99.2%). TMM with a QL F-test has a slightly higher specificity (28%) than RLE/UQ (24.5%). Although a t-test for DEGs analysis in RNA-seq studies is not commonly used due to the distribution of the read counts in RNA-seq data following a negative binomial [26, 49], the voom-limma package has been recently proposed [29] and was reported to have good control of FDR, but low power for small sample size [36, 37]. Therefore, it is interesting to examine the results from a t-test using log-transformation of read counts following one of the four normalization methods. As expected, the results show there is no difference between the UQ and UQ-pgQ2 methods since the median scaling factor estimated for each gene across samples in UQ-pgQ2 was canceled while applying a t-test [50]. Although UQ/UQ-pgQ2 performed relatively better than TMM and RLE, with a specificity of 48.7%, there was a tradeoff with lower power of 93.1%, consistent with previous reports [36, 37]. The results also suggested a t-test is not a better choice for the TMM and RLE methods compared to other tests such as a Wald test or an exact test/QL F-test.

Overall, for this comparison study of the four test statistics (the exact test/QL F-test, Wald test and t-test), the results from MAQC2 data demonstrated that UQ-pgQ2 and TMM combined with an exact test/Wald test performed much better than using a QL F-test and t-test in terms of sensitivity/power and specificity/FDR while UQ and RLE were varied.

The results from an additional analysis of MAQC3 with five replicates had similar conclusions for UQ-pgQ2 normalization (Table 2). Briefly, UQ-pgQ2 with an exact test was the best choice and achieved the highest specificity among the four normalization methods for all four test statistics. The results also show that all normalization methods combined with a t-test/voom-limma achieved better specificity than a Wald test and QL F-test while all the methods have a sensitivity close to or above 90% with a tradeoff of lower power than others. Thus, the results using MAQC3 data suggested that an exact test for UQ-pgQ2 or a t-test from voom-limma seems to control the FDR better than other methods when sample sizes or replicates are relatively large.

Finally, the results from the analysis of both MAQC datasets suggested the four normalization methods combined with the three test statistics (exact test, QL F-test and Wald test) can achieve a great sensitivity/power while a t-test from voom-limma has relatively lower power with unstable performance on the control of FDR. Although the UQ-pgQ2 method performed relatively better for controlling FPs, all normalization methods have a problem maintaining the actual FDR below the nominal level of 0.05, which agrees with previous reports [18, 23, 33].

### Within-group analysis of real cancer datasets for detecting FPs given a desired sample size

**Fig. 1:**
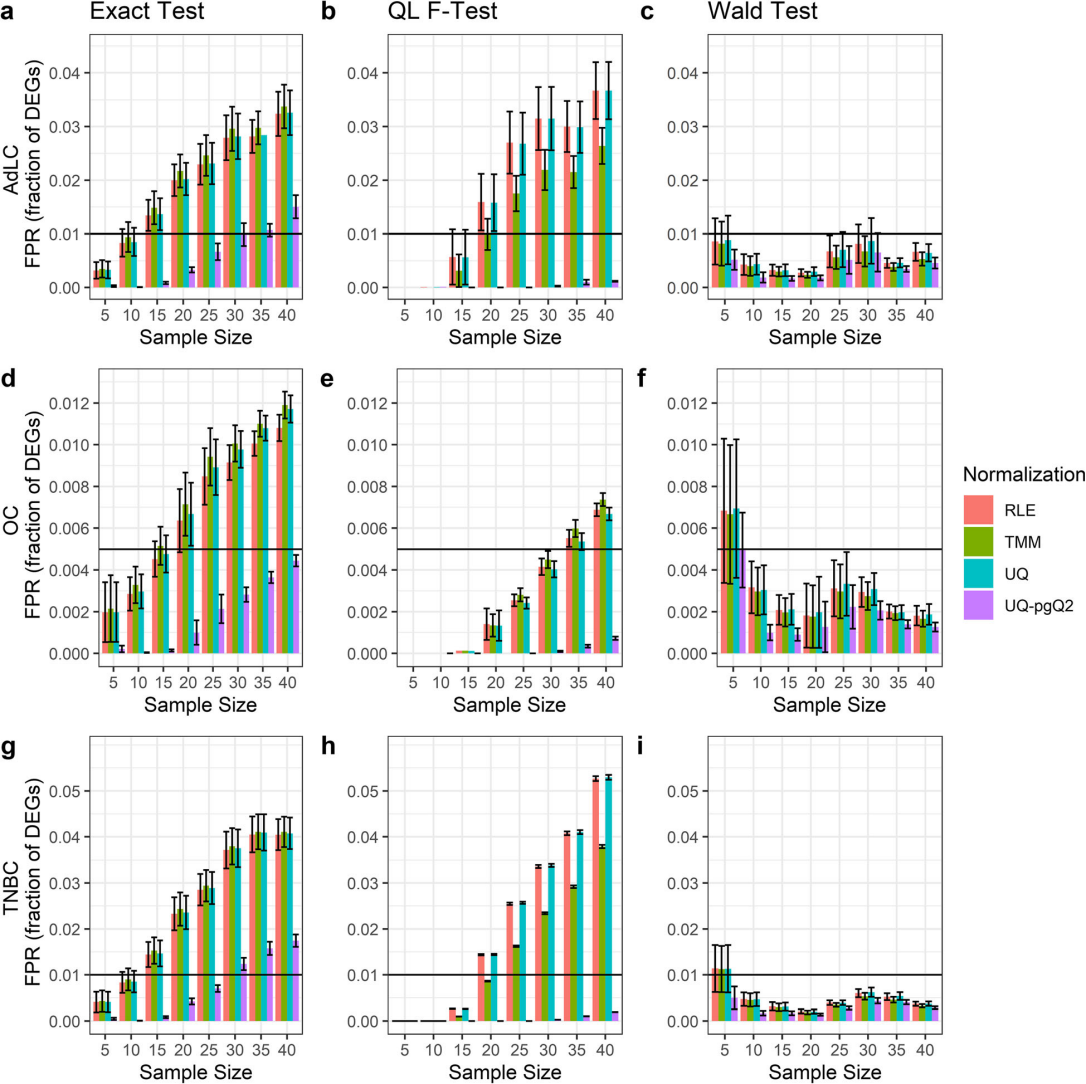
False positive rates estimated via intra-group analysis of AdLC, OC and TNBC data. Illustrated are the fractions of FPs estimated from the RLE (pink), TMM (green), UQ (blue) and UQ-pgQ2 (purple) normalization using the exact test, QL F-test or Wald test for sample sizes of 5, 10, 15, 20, 25, 30, 35 and 40. The plots are based on AdLC data (**a**-**c**), OC data (**d**-**f**) and TNBC data (**g**-**i**)

A type I error rate and FDR are the most important performance measures for evaluating DEG analysis methods. The large number of replicates from TCGA human cancer datasets including non-small cell lung cancer with adenocarcinoma subtype (AdLC), ovarian cancer (OC) and triple negative breast cancer (BC) allows us to perform within-group analysis of FPs for estimation of a type I error rate. The four normalization methods (TMM, RLE and UQ and UQ-pgQ2) combined with the exact test, QL F-test or Wald test were compared given a desired sample size of replicates in a single group. The two synthesized groups with an equal and desired sample size were randomly subsampled from the same cancer subtype. Under the null hypothesis, the genes between the two synthesized groups in this study are not expected to be differentially expressed. Thus, the DE genes identified are defined as FPs. Given a FDR cutoff of 0.05 and an absolute value of FC cutoff at 2 as a conventional way for identifying DEGs, the FPR (a fraction of DEGs) and the number of FPs identified are illustrated in Fig. 1 and Additional file 1: Figure S1, respectively. Although the FPR for all the four normalization methods based on the three datasets are below 0.05, the performance of these methods are significantly different.

First, using an exact test/QL F-test, we found that the FPR in Fig. 1 increases as the sample size for all the methods increases from 5 to 40 in the three cancer datasets (Fig. 1a, b, d,e, g and h). However, not unexpectedly, this pattern was not observed when a Wald test was used. With a Wald test, higher FPRs are observed when sample sizes are five and they tend to decrease at larger sample sizes of 10, 15, 20, 25, 35 and 40 (Fig. 1c, f and i), but the FPR for different sample sizes varies (Fig. 1c).

Second, we found that the exact test at a sample size of five can achieve a smaller value of FPRs than a Wald test for all the methods (RLE in pink, TMM in green, UQ in blue and UQ-pgQ2 in purple). This suggests that when a sample size is small, an exact test is more conservative than a Wald test*.* Moreover, the QL F-test combined with any of four normalization methods at sample sizes of 5, 10 and 15 can achieve the smallest FPR compared to the other two tests (Fig. 1b, e and h). However, when a sample size becomes large (*n* > 15), a Wald test for RLE, TMM and UQ is more conservative than choosing the exact test or QL F-test.

Third, in this study, the differences among RLE, TMM and UQ normalization methods are relatively small and varied. We found that the two-step normalization method UQ-pgQ2, consistently performed better than the others by achieving the smallest FPR and number of FPs given a desired sample size in all scenarios (Fig. 1 and Additional file 1: Figure S1). Overall, the results illustrate that a QL F-test with a UQ-pgQ2 may be the best option for DEG analysis when FDR is more important to be considered. These observations are consistent for the three real datasets.

### The effect of sequencing read depth of OC samples on the analysis of FPs from the normalization methods and test statistics

**Fig. 2:**
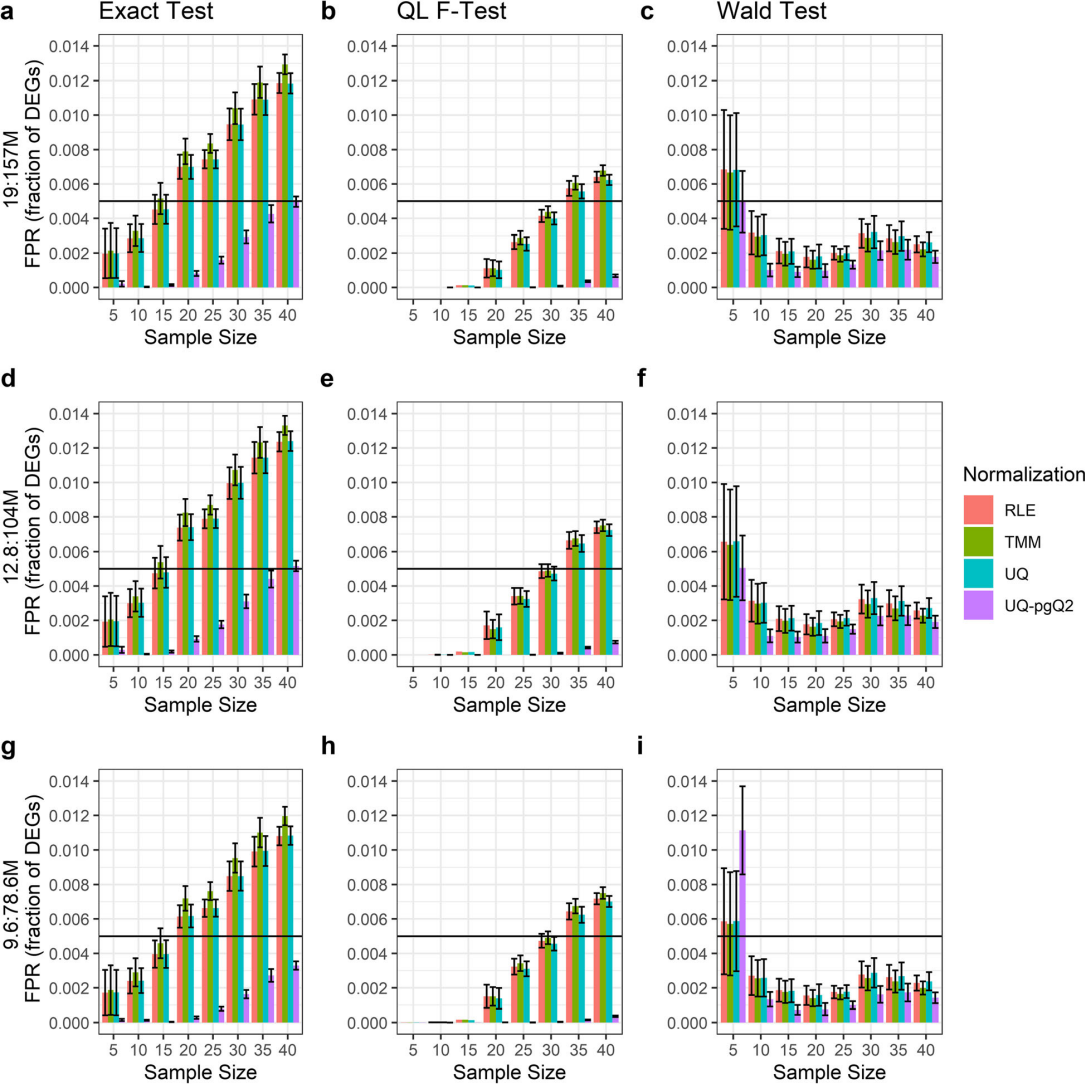
False positive rates estimated via intra-group analysis of 379 OC samples with different read depths. Illustrated are the fractions of FPs estimated from the RLE, TMM, UQ and UQ-pgQ2 normalization with the exact test, QL F-test or Wald test for sample sizes of 5, 10, 15, 20, 25, 30, 35 and 40. The plots are based on a read depth from 19 to 157 million (**a**-**c**), from 12.8 to 104 million (**d**-**f**), and from 9.6 to 78.6 million (**g**-**i**)

**Fig. 3:**
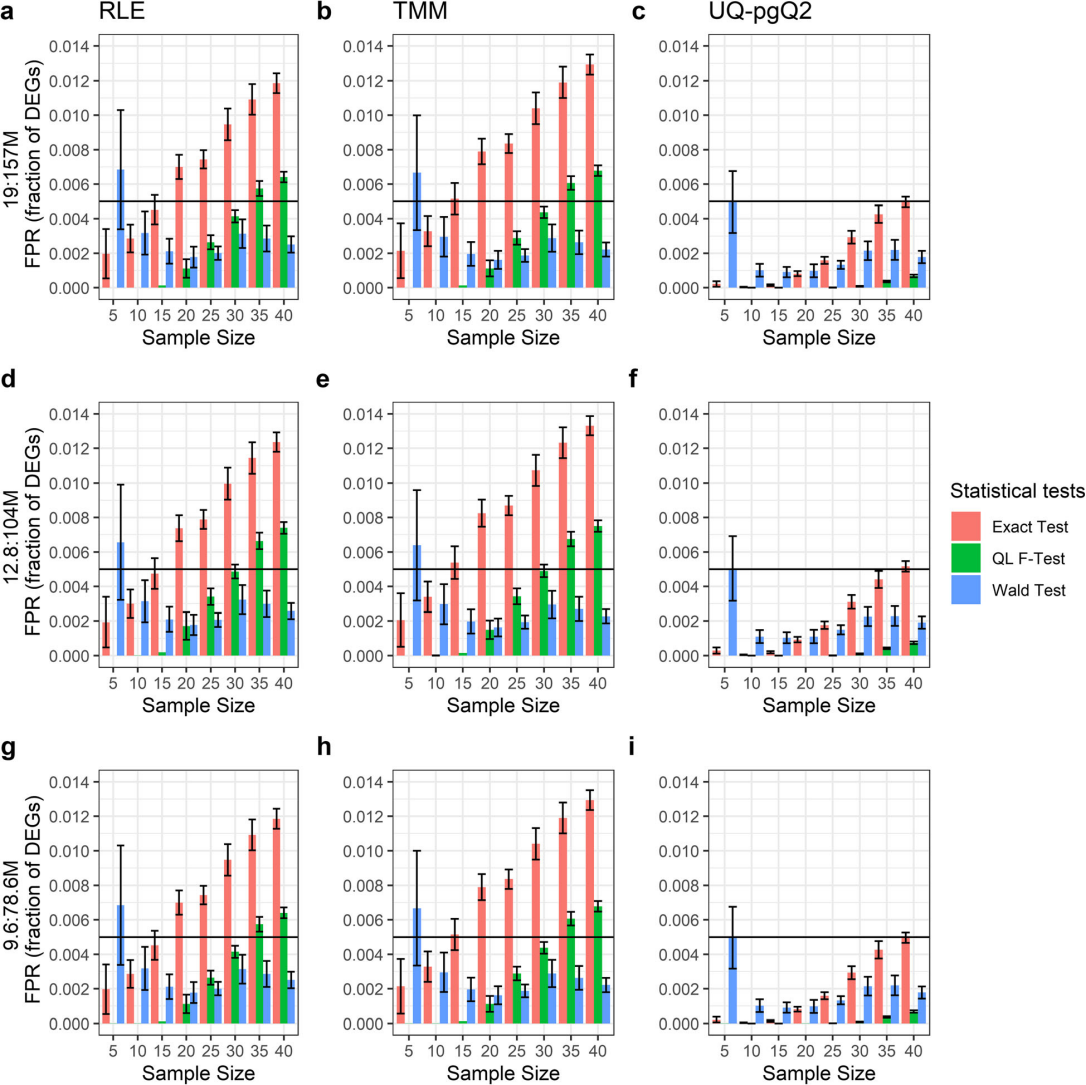
False positive rates estimated from three test statistics via intra-group analysis of OC samples. Illustrated are the fractions of FPs estimated from the exact test/QL F-test and Wald test combined with the RLE, TMM and UQ-pgQ2 normalization methods, based on the read depths from 19 to 157 million (**a**-**c**), from 12.8 to 104 million (**d**-**f**) and from 9.6 to 78.6 million (**g**-**i**)

Next, we examined whether the read depth in a RNA-seq study affects the number of FPs for the normalization and test statistical methods given a desired sample size. The read depths of the 379 OC samples range from 19 to 157 million reads. Two new datasets were generated by simply reducing the read depth of the OC samples to one-third or half. Thus, we obtained one dataset with the read depth in the range of 12.8 to 104 million reads and the other with a range of 9.6 to 78.6 million reads. Given an FDR cutoff of 0.05 and an absolute FC cutoff of 2, the FPR estimated from the number of FPs identified in Additional file 1: Figure S2 and S3 is illustrated in Figs. 2 and 3.

Within-group analysis of three sets of data with different read depths revealed that the FPR from higher read depths (19-157 M and 12.8-104 M) in Fig. 2a to 2f is slightly larger than those with smaller read depths (9.6–78.6 M) in Fig. 2g to 2i regardless of normalization methods and sample sizes. However, the difference for the samples with the read depths between 19 and 157 M and 12.8-104 M (a smaller change) is varied and very small. Regardless of read depth, similar patterns are observed between Figs. 1 and 2. Overall, UQ-pgQ2 method is more conservative than the others in most of scenarios given a desired sample size and statistical test. However, Fig. 2 shows that UQ-pgQ2 combined with a Wald test in read depth of 9.6 to 78.6 million is more liberal at the sample size of five.

Figure 3 and Additional file 1: Figure S3 further demonstrate the difference between three test statistics (the exact test in pink, QL F-test in green and Wald test in blue) and three normalization methods. The FPR increases as sample sizes increase while using the exact test and QL F-test, but the impact of FPR by the sequencing read depth are very small. For a Wald test, the FRP is larger than the one from other tests when the sample size is five. Moreover, the FPR from RLE and TMM combined with the three tests are similar (Fig. 3a, b, d, e, g and h). In contrast, UQ-pgQ2 from the three tests (Fig. 3c, f and i) can achieve lower FPR compared to other normalization methods.

### The effect of sequencing read depths from simulated data on the analysis of FPs given a desired sample size

**Fig. 4:**
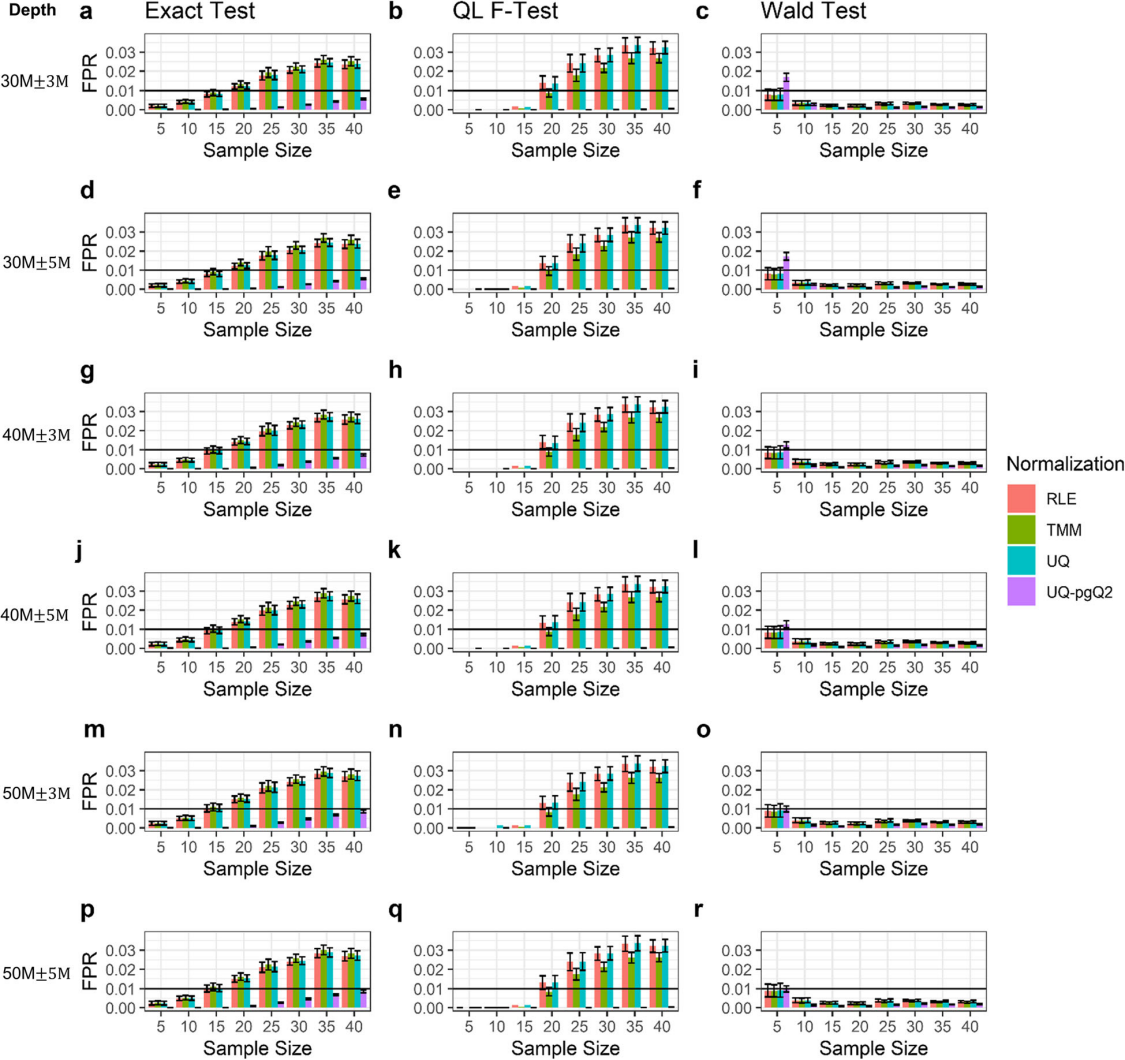
False positive rates estimated from simulated data with variable read depths given fixed sample sizes. Illustrated are the fractions of FPs estimated from the RLE, TMM, UQ and UQ-pgQ2 normalization methods using the exact test, QL F-test or Wald test for sample sizes of 5, 10, 15, 20, 25, 30, 35 and 40. Plots are based on 122 simulated data with the mean read depth of 30 million and SD of 3 (**a**-**c**) or 5 million (**d**-**f**); a mean read depth of 40 million and SD of 3 (**g**-**i**) or 5 million (**j**-**l**); a mean read depth of 50 million reads and SD of 3 (**m**-**o**) or 5 million (**p**-**r**)

Each of the six simulated datasets contains 122 samples with a desired mean read depth of 30, 40 and 50 million reads with a standard deviation (SD) of 3 and 5 million reads, respectively. In this study, we examined whether the simulated data with a desired read depth and SD affects the number of FPs/FPRs from different normalization and test statistical methods given a desired sample size. Given a FDR cutoff of 0.05 and an absolute value of FC cutoff at 2, the results are illustrated in Fig. 4 and Additional file 1: Figure S4**.**

Overall, the results from the simulated data are similar to the ones illustrated in Figs. 1 and 2. When the read depths increase from 30 to 50 million reads (Fig. 4a, d, g, j, m and p), the FPR from an exact test slightly increases, which is consistent with the observation from real data (Fig. 1). But, these patterns were not observed when using the Wald test and QL F-test. We also observed that UQ-pgQ2 (purple) performed the best with the lowest FPR while TMM (green) performed the worst with the largest FPR using an exact test. For a Wald test, UQ-pgQ2 performed the worst for a sample size of five and performed the best for sample sizes of 10 or larger while the other methods performed similarly. For a QL F-test, all the methods perform similarly by achieving very small FPR for a given sample size of 5, 10 and 15. When the sample size is 20 or larger, similar results are observed except TMM combined with the QL F-test can achieve a smaller FPR than RLE and UQ methods. Taken together, with a QL F-test, UQ-pgQ2 outperformed the other methods by achieving the smallest value of FPR regardless of the sample sizes and sequencing read depth.

### Between-group analysis for identifying DEGs from cancer samples versus the normal controls given a desired sample size

**Fig. 5:**
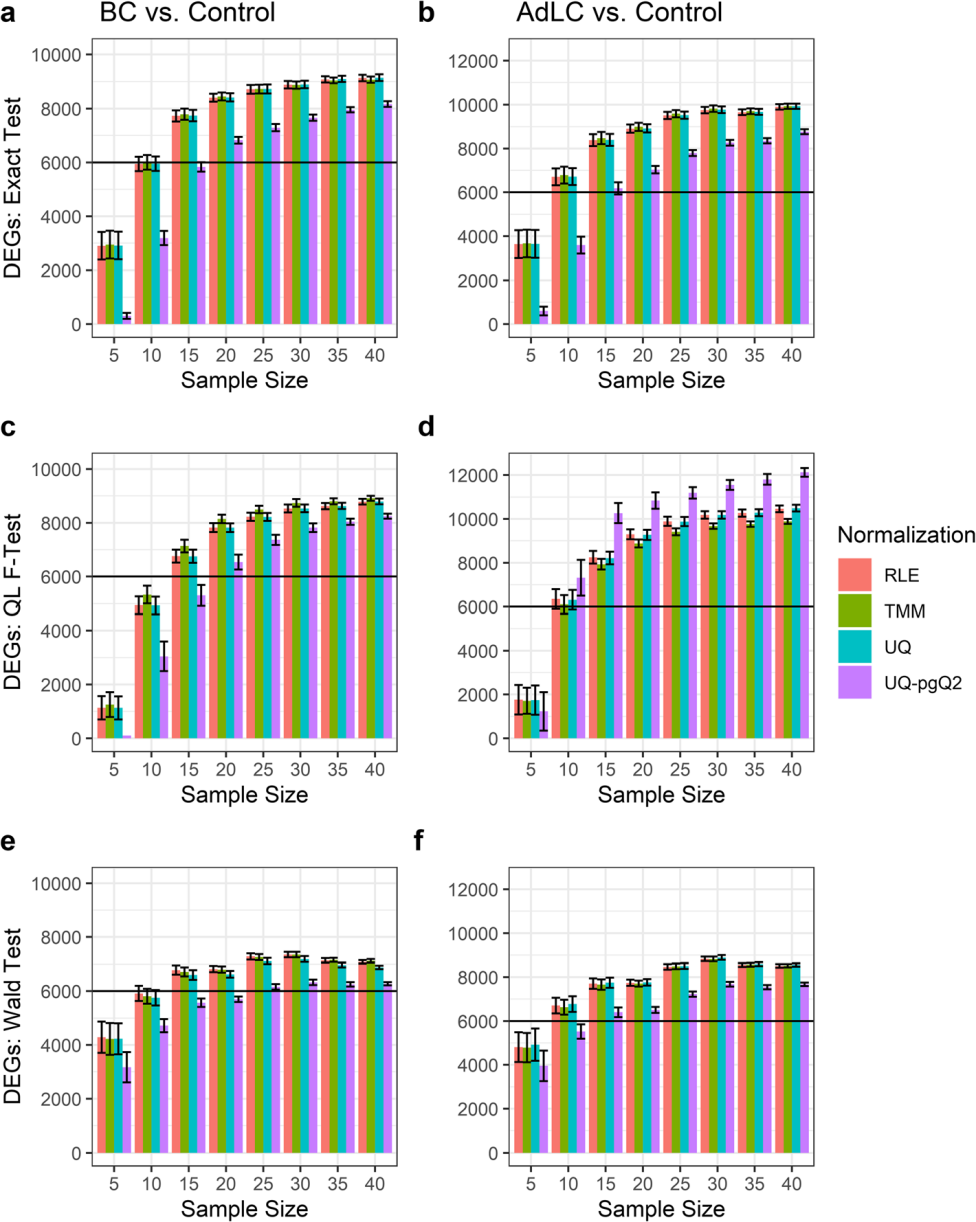
DEGs identified from the four normalization methods for a balanced two-groups comparison. Illustrated are the number of DEGs identified in 117 TNBC and 112 normal control samples (**a**, **c**, **e**), and 535 AdLC and 59 normal control samples (**b**, **d**, **f**). Analysis was accomplished using the exact test (**a**, **b**), QL F-test (**c**, **d**) and Wald test (**e**, **f**) listed

Finally, we evaluated the four normalization methods with three test statistics based on the number of significant DEGs identified using the paired BC data (117 BC and 112 control) and lung cancer data (535 AdLC and 59 normal control). Given a desired sample size, the cancer and control groups were subsampled from BC/AdLC and their control samples for 50 times, respectively. The number of DEGs detected from each method with a desired sample size is the average of the number of DEGs while bootstrapping for 50 times. Given an FDR cutoff of 0.05 and an absolute FC cutoff of 2, the number of DEGs identified is illustrated in Fig. 5.

First, we found that the number of DEGs increases as the sample size increases from 5 to 30 for each method (Fig. 5a-f). As the sample size increases from 30 to 40, the number of DEGs varies either slightly increasing (Fig. 5a-d) or decreasing (Fig. 5e, f).

Second, given sample sizes of 15 or more, an exact test combined with one of the four normalization methods detected more DEGs than the QL F-test and Wald test in the most scenarios, which indicates that the Wald test and QL F-test are more conservative than the exact test while the exact test has more power than others. However, given a sample size of five, the Wald test can identify more DEGs than the exact test and QL F-test.

Third, comparing the four normalization methods, UQ-pgQ2 (purple) is more conservative than the others in the most scenarios except the QL F-test for AdLC data, where UQ-pgQ2 achieved the highest detection power for given sample sizes of 10 or larger (Fig. 5d). However, given a sample size of five, the number of DEGs detected from UQ-pgQ2 combined with the exact test or QL F-test is small (Fig. 5a-c). This observation suggests that RLE, TMM and UQ combined with the exact test or QL F-test from *edgeR* are the better choice for achieving a better detection power than UQ-pgQ2 for a small sample size such as five and the number of DEGs identified from UQ-pgQ2 looks more reasonable given a sample size ≥10. Since we do not know the number of true positives and true negatives, we assume the method detecting the highest number of DEGs may have the highest sensitivity or detection power.

## Discussion

Some previous studies comparing normalization methods have reported that both *DESeq2* and *edgeR* with an exact test/likelihood ratio test failed to maintain the actual FDR below the nominal level of 0.05, suffering from being” oversensitive” in some cases [14, 18, 23, 37, 51]. Our recently proposed normalization method, UQ-pgQ2, combined with an exact test had a better performance than the others in terms of controlling the type I error rate and FDR. However, in that previous study, sample sizes (replicates) for a two-group comparison were smaller than six in many cases. Currently, common sample sizes in RNA-seq studies can range from a minimum of 3 up to several hundreds of biological replicates. It is known that a Wald test can be used for testing a hypothesis of a parameter when sample sizes are usually larger than 30 based on an asymptotic theorem. Recently, *edgeR* provided a QL F-test which was recommended for studies with a small number of replicates in RNA-seq data. A study of single-cell differential expression analysis observed that edgeR/QLF performed well after filtering out lowly expressed genes [52]. Thus, a comprehensive comparison of QL F-test and other test statistics combined with newly developed normalization methods (UQ-pgQ2 or voom-limma) for DEG analysis of bulk RNA-seq data deem to be needed. Furthermore, one outstanding question is whether the Wald test, QL F-test or t-test combined with a normalization method performs better than an exact test used in the previous study in terms of controlling FDR. Another question is what is the best combination between normalization and test statistical methods for the control of type I error rate given a desired sample size. To address these issues, we focused on four normalization methods and three test statistics using both real RNA-seq datasets and simulated data given sample sizes at 5, 10, 15, 20, 25, 30, 35 and 40.

Initially, we used two benchmark MAQC datasets for the DEGs analysis. The results from MAQC datasets show that UQ-pgQ2 combined with an exact test can achieve the highest specificity with the sensitivity higher than 90%. We also observed that *edgeR* with TMM normalization performed better than RLE and UQ with the Wald test or QL F-test in terms of sensitivity and specificity using the small samples sizes of MAQC datasets.

Next, we used an intra-group approach to calculate the number of FPs and FPR from null datasets generated from real data and simulated data. We compared these methods by taking into consideration of sample sizes and read depths. The results from this approach have shown that in general the QL F-test combined with any one of four normalization methods (RLE, TMM, UQ and UQ-pgQ2) performs the best for achieving the lowest value of FPRs when the sample size is small (≤15). However, when the sample size is large, UQ-pgQ2 combined with the QL F-test performs the best and the Wald test performs much better than an exact test and QL F-test with FPR below 0.01. Moreover, we found the read depths from simulated data with 30 to 50 million reads have a minimal impact on detection power and FDR. Furthermore, comparing DEG analysis of BC and AdLC suggests that the RLE, TMM and UQ combined with an exact test or a Wald test have higher sensitivity or power than the UQ-pgQ2 method. However, these methods may suffer oversensitivity when the sample sizes are large. In addition, the results from AdLC show UQ-pgQ2 combined the QL F-test achieves higher detection power than other combined methods, but this observation is not consistent with the one from BC datasets.

Furthermore, it is important to note that the evaluated methods may not be applicable to all type RNA-seq data. For example, single-cell RNA sequencing (scRNA-seq) data has been increasingly used to assess different cell states and cell types such as stem cell, neuron cell and cancer cells [53–55]. A DEG analysis between different cells in scRNA-seq can help to uncover driver genes in cancer research [56]. Due to technical limitations, scRNA-seq data generally have low library sizes resulting in a large fraction of ‘dropout’ events as well as huge heterogeneity, which introduces a major challenge in identification of DEGs. Given the special characteristics, many new methods have been developed especially for DE analysis of scRNA-seq data [57–60]. A few studies have compared DEG analysis tools for scRNA-seq data and found that existing methods for analysis of bulk RNA-seq data perform as well as, or not worse than, those specifically developed for scRNA-seq data in terms of the power and FDR [52, 61]. A recent study has conducted DEG analysis for a comparison of eleven tools and two of them (edgeR and DESeq2) were designed originally for bulk RNA-seq [62]. In this study, a high disagreement among these tools in calling DEGs were identified due to a trade-off between the sensitivity and FDR. It also reported that current methods developed for scRNA-seq did not show better performance compared to *edgeR* and *DESeq2*, which is consistent with the findings from previous studies. Thus, it is reasonable for us to mention here whether the newly developed normalization methods such as UQ-pgQ2 and Med-pgQ2 can be used for DEG analysis in scRNA-seq data for a control of FDR.

Finally, our study has some limitations. First, this work is limited to gene-level analysis and balanced designs. Second, the per gene normalization method, UQ-pgQ2, is only used for the DEG analysis among the groups and is not applicable for comparing genes within a group. Third, we used within group analysis of several cancer subtypes to identify DEGs and we assumed the DEGs as false positives that were used to calculate FPRs. Although this approach has been used to estimate the type I error or FPs for the comparison of normalization and test statistical methods in several studies [32, 37, 46], these identified genes may contain some TPs due to the variation between cancer patients. However, this limitation can be offset by the two benchmark MAQC RNA-seq data. Finally, since voom-limma with a t-test used for DEGs analysis in bulk RNA-seq data, we need to address here that per gene normalization in UQ-pgQ2 would not alter the DEG results due to the invariant property of t-test for the linear transformations of gene counts across samples.

## Conclusions

Taken together, we found the UQ-pgQ2 method with an exact test is the best choice for DEG analysis in terms of controlling false positives when using the benchmark MAQC datasets. However, based on an intra-group analysis of real data and simulated data, we found UQ-pgQ2 combined with a QL F-test outperforms other methods by achieving the smallest value of FPR, and a Wald test from *DESeq2* can achieve a FPR below 0.05 when sample sizes are large. We observed that the RLE, TMM and UQ normalization methods combined with the Wald or exact test/QL F-test performed similarly and read depths have minimal impact on detection of DEGs from the analysis of simulated data. We hope this new finding can serve a guide for researchers to properly choose the normalization and test statistical methods for identifying DEGs while taking sample sizes into the consideration. As scRNA-seq technology emerges, UQ-pgQ2 combined with the QL F-test may be useful for DEG analysis of scRNA-seq data for controlling FDR, but an evaluation should be conducted in the future.

## Methods

### Data sources

#### Microarray quality control project (MAQC) RNA-seq data

Two benchmark RNA-seq datasets (MAQC2 and MAQC3) were used for evaluation [18, 34, 46]. These datasets have two conditions: human brain reference RNA (hbr) and universal human reference RNA (uhr). MAQC2 RNA-seq data contains two replicates in each condition (hbr1, hbr2, uhr1 and uhr2). The hbr1 / uhr1 and hbr2 / uhr2 samples were prepared and sequenced in different labs. MAQC3 RNA-seq data contains five replicates in each condition.

#### TaqMan qRT-PCR data

In the MAQC project, a benchmark PCR dataset of 1044 genes was used for validation. Detailed information for processing and analyzing this data has been previously described [17, 18, 38, 47]. Briefly, we identified 388 genes as true positives and 143 genes as true negatives to evaluate these methods.

#### Human cancer RNA-seq datasets from TCGA

Three types of human BC, OC and LC data were downloaded from TCGA website [48]. In this study, we used one subtype from each cancer type. The 122 TNBC, 379 OC, 523 AdLC and 59 normal control samples were extracted using an R script (v3.5.2). For DEG analysis, 117 BC and 112 paired normal control samples were extracted. Lowly expressed genes in each TCGA dataset were filtered out when the zero counts across samples were greater than 50% across the samples. The remaining genes were used for the downstream analysis. These datasets are available in Additional file 2.

### Simulated data using Monte Carlo method

The simulated data is based on the 122 TNBC samples with a range of total read depth between 23.8 and 98.7 million reads. For our simulation, let *G* be the total number of genes (*G* = 30,831) with desired sample sizes (*n* = 5, 10, 15, 20, 25, 30, 35 and 40) denoted as the number of replicates. Let *R*_*ig*_ be the read counts in sample *i* and gene g and *N*_*i*_ be the total number of reads (read depth) in sample *i* as estimated from the 122 TNBC samples. Let *p*_*ig*_ be the proportion for the gene g in sample *i*, where ${\hat{p}}_{ig}$ was estimated from *R*_*ig*_ divided by *N*_*i*_. For simulating read depths, we modeled the independent and desired read depth *N*_*i*_ (*i* = 1, …, *n*_*i*_) to follow a normal distribution with a mean of 30, 40 and 50 million reads, and standard deviation of 3 million reads for scenario one and 5 million reads for scenario two for each *N*,. The resulting distributions are *N*_*i*_ ∼ *N* (30, 3^2^) and *N*_*i*_ ∼ *N*(30, 5^2^); *N*_*i*_ ∼ *N*(40, 3^2^) and *N*_*i*_ ∼ *N*(40, 5^2^); and *N*_*i*_ ∼ *N*(50, 3^2^) and *N*_*i*_ ∼ *N*(50, 5^2^), respectively.

As an example, in the case of generating samples with a mean read depth of 30 million reads and a standard deviation of 3 million reads given a desired sample size of five, first, ten samples for the two groups were randomly selected from the set of 122 TNBC cases. We estimated ${\hat{p}}_{ig}$ for each sample and generated read depths based on *N*_*i*_ ∼ *N*(30, 3^2^) using the Monte Carlo method. Finally, the raw counts for each sample were generated using multinomial distribution given a desired *N*_*i*_ and a vector of ${\hat{p}}_{ig}$ for sample *i*. This procedure was repeated 50 times given desired sample size of 5, 10, 15, 20, 25, 30, 35 or 40 combined with *N*_*i*_ ∼ *N*(30, 3^2^). Thus, the data in the two groups was generated in the scenario of *N*_*i*_ ∼ *N*(30, 3^2^).

### Sequence alignment and extraction of gene counts

The raw Sequence Read Archive (SRA) files for the MAQC2 and MAQC3 with two conditions were converted to .fastq files and then aligned to the human hg38 reference genome using STAR (v2.6.0c) [11] and the Ensembl hg38 annotation gtf file (GRCh38.82). The mapped counts for 60,483 genes per sample were extracted using HTSeq-scripts-count (version 2.7.5). Lowly expressed genes with zero counts across all the samples were further filtered. The MAQC datasets are available in Additional file 2.

### Normalization methods and software packages

The normalization methods, statistical tests and software packages for the DEG analysis between the two-group comparisons are summarized in Table 3. The R code used in our analysis is available in Additional file 3.

**Table 3:** Summary of normalization methods combined with statistical tests in software packages used

Normalization method	Description of normalization	Distribution	Exact test	Wald test
RLE	Per sample by Relative Log Expression	NB	*edgeR* (v3.24.3)	*DESeq2* (v1.22.2)
TMM	Per sample by Trimmed Mean M-values	NB	*edgeR* (v3.24.3)	*DESeq2* (v1.22.2)
UQ	Per sample scaled by upper quantile	NB	*edgeR* (v3.24.3)	*DESeq2* (v1.22.2)
UQ-pgQ2	Per sample scaled by upper quantile and per gene by medium across samples	NB	*edgeR* (v3.24.3)	*DESeq2* (v1.22.2)

*NB* negative binomial distribution

#### Normalization methods

Four methods (RLE, TMM, UQ and UQ-pgQ2) were used to normalize RNA-seq data. In this study, RLE and TMM normalizations were implemented in *DESeq2* and *edgeR,* respectively [25, 63]*.* UQ and UQ-pgQ2 were implemented using an R script [18, 23, 28]*.* UQ-pgQ2 uses a two-step approach for normalization [18]. Briefly, assuming *G* genes and *m* samples, the scaling factor for UQ normalization is calculated from the 75th percentile (Q3) of the counts for each sample after removing genes with zero counts. Gene g in sample *j* is scaled by the UQ scaling factor and then multiplied by the mean of the scaling factors from *m* samples. Therefore, ${X}_{gj}^{UQ}$ is gene g in sample j with UQ normalized counts. Then, UQ-normalized gene *g* is further scaled by its median (Q2) of read counts across m samples and then multiplied by 100. Therefore, ${X}_{gj}^{UQ- pgQ2}$ is gene *g* in sample *j* and normalized by UQ-pgQ2 method.

#### Software packages and test statistics used for DEGs analysis

The exact test, QL F-test and Wald test were used for the detection of DEGs. In this study, we used the exact test and QL F-test implemented in *edgeR (v3.24.3)* [45]. A Wald test implemented in *DESeq2 (v1.22.2)* was reported to improve the sensitivity compared with an exact test implemented in *DESeq* [25].

#### Log-transformed Wald test in a NB distribution

The Wald statistical test is an asymptotic test based on the normal approximation, which utilizes the large-sample properties of maximum likelihood estimation (MLE). In *DESeq2*, the read counts *K*_*ij*_ in gene *i* sample *j* is modelled by a generalized linear model (GLM) of the NB family with a log link:

*K*_*ij*_~*NB*(*mean* = *u*_*ij*_, *Dispersion* = *α*_*i*_), *u*_*ij*_ = *s*_*ij*_*u*_*ij*_ and log ${q}_{ij}=\sum r{x}_{jr}{\beta}_{ir}$. The *s*_*ij*_ = *s*_*j*_ is the size factor used to normalize the gene read counts in sample *j* and the *q*_*ij*_ is the true expression of gene *i* [25].

For a GLM with two conditions and a single gene in sample j, the log-transformed Wald test has been described in several studies [64–66]. Briefly, the treatment and control group indicators (*x*_*r*_) take the value 1 and 0, respectively, resulting $\frac{q_1}{q_0}={e}^{\beta_1}$ . For the differential expression gene analysis, the ratio ρ =*q*_1_/*q*_0_ (a fold change) is used for a hypothesis test. Testing the hypothesis of *q*_*r*_: *H*_0_: *ρ* = 1 vs. *H*_1_: *ρ* ≠ 1 is equivalent to *H*_0_: *β*_1_ = 0 vs. *H*_1_: *β*_1_ ≠ 0 in GLM. The two-sided Wald test is defined as $\mid {Z}_w\mid =\frac{\left|{\hat{\beta}}_1\right|}{\sqrt{Var\left({\hat{\beta}}_1\right)}}$, where the $Var\left({\hat{\beta}}_1\right)$ is estimated from the variance-covariance matrix of (*β*_0_ ,*β*_1_), which is the inverse of the Fisher information matrix of ${I}_{n_0{n}_1}\left({\beta}_0\ \mathrm{and}\ {\beta}_1\right)$ asymptotically. To reject the null hypothesis, $\left|{Z}_w\right|>{Z}_{1-\frac{\alpha }{2}}$ is defined. Thus, this gene with *p*-value less than 0.05 is called a DEG. For testing multiple genes simultaneously, the *p*-values are further corrected using Benjamini-Houchberg FDR method [67].

#### Exact test in a NB distribution

Since RNA-seq data are read counts, an exact test has been implemented similarly in *DESeq* and *edgeR* [26, 68]. For a gene in a two-group comparison, the exact test has been described by several studies [18, 26, 69] . Briefly, *Y*_*ij*_ is denoted the normalized read counts of the single gene in condition *i* = A and B, and replicate *j* = 1, …, *n*_*i*_*.* Then, the distributions of ${Y}_{ij}\ \mathrm{and}\ \sum \limits_j^{n_i}{Y}_{ij}$ are assumed to follow a negative binomial distribution with an expected mean *u*_*i*_ and dispersion *ϕ* expressed as:

*Y*_*gij*_~*Y*_*gij*_~*NB*(*u*_*gi*_, ϕ), $\sum \limits_j^{n_i}{Y}_{ij}\sim NB\left({n}_i\cdotp {u}_i,\frac{\upphi\ }{n_i}\right),E\left(\sum \limits_j^{n_i}{Y}_{ij}\right)={n}_i\cdotp {u}_i$, and Var $\left(\sum \limits_j^{n_i}{Y}_{ij}\right)={n}_i\cdotp {u}_i+{n}_i\cdotp {u}_i^2\cdotp \upphi$. The null hypothesis *H*_0_ : *u*_*A*_ = *u*_*B*_ is to identify DEGs between conditions A and B and the total normalized counts in each condition are ${Y}_A=\sum \limits_j^{n_A}{Y}_{Aj}$ and ${Y}_B=\sum \limits_j^{n_B}{Y}_{Bj}.$ The total counts of two conditions for the gene are *Y*_*S*_ = *Y*_*A*_ + *Y*_*B*_.

Since *Y*_*A*_ and *Y*_*B*_ are assumed to be independent, the joint probability of *P*(*Y*_*A*_ = *y*_*A*_, *Y*_*B*_ = *y*_*B*_ ) under *H*_0_ is *P*(*Y*_*gA*_ = *y*_*gA*_) × *P*(*Y*_*gB*_ = *y*_*gB*_). Thus, the *p*-value from an exact test [26] is calculated by summation of the probability of a pair of *P(a, b)* that is less than or equal to the observed *P(y*_*A*_, *y*_*B*_*)* given that the overall summation of *P(a, b)*. The pair of variables *a* and *b* are defined as *a* = 0, …, *Y*_*S*_ *and b* = *Y*_*S*_ − *a*. Then the p-value for gene *g* is

*p*. *value* = *P*_1_/*P*_2_, where

$$ {P}_1={\sum}_{\begin{array}{c}a+b={Y}_s\\ {}P\left(a,b\right)\le P\left({y}_{A,}{y}_B\right)\end{array}}P\left({Y}_A=a\right)\times P\left({Y}_B=b\right), $$

and

$$ {P}_2={\sum}_{a+b={Y}_S}P\left(a,b\right). $$

The p-value is further adjusted for multiple test correction using the Benjamini-Hochberg FDR methodology. A study reported that the exact test performed better for achieving a smaller FDR than a Wald and likelihood ratio tests when the sample size is small [49].

### Intra-group analysis to identify the number of FPs given a desired sample size

To compare the methods, we utilized the desired number of replicates (5, 10, 15, 20, 25, 30, 35 and 40) to estimate the number of false positives and the corresponding FPR, a fraction of DEGs via intra-group analysis. Given sample sizes, two groups are generated by randomly subsampling from a single cancer group. Since the samples originate from the same condition, the number of DEGs expected in such two-group comparisons should be zero. Thus, by this assumption, any DEGs would be defined as false positives that were further used for estimating FPR under a null hypothesis [32, 37, 52, 70].

### Bootstrap differential expression analysis of cancer versus normal control samples given a desired sample size

Given desired sample sizes of 5, 10, 15, 20, 25, 30, 35 and 40, the two-group data (cancer and normal control) were randomly subsampling from BC/AdLC and the normal samples, respectively. Subsequently, the cancer and control groups were normalized by one of the four normalization methods used in this study. The DEG analysis of the normalized data was performed with the aid of *edgeR*, *DESeq2* and *limma* tools. The results from these packages include the log_2_ transformed fold change, *p*-values and Benjamini-Hochberg FDRs. In this study, we defined significant DEGs using an absolute value of log_2_ FC cutoff at one (|FC| = 2) and a FDR cutoff at 0.05. The bootstrapping process of running each DEG algorithm was iteratively repeated 50 times. The mean number of DEGs corresponding to the standard error was imputed from the 50 iterations.

## Supplementary information

**Additional file 1: Figures S1-S4.** Illustrated the number of false positive genes identified from intra-group analysis corresponding to Figs. 1, 2, 3 and 4, respectively.

**Additional file 2.** Contains datasets used for the analysis. This zipped file folder contains MAQC2 and MAQC3 raw read counts, cancer raw data files filtered with zero counts (AdLC, OC and TNBC), and description of these data files named Supplementary Material.docx.

**Additional file 3.** R code for the analysis.

## Data Availability

The datasets and R code used in this study are available in Additional files 2 and 3.

## Abbreviations

AdLC: Human non-small cell lung cancer with adenocarcinoma subtype
BC: Human breast cancer
DE: Differential expression
DEGs: Differentially expressed genes
FC: Fold change
FDR: False discovery rate
FP: False positive
FPKM: Fragments Per Kilobase per Million mapped fragments
FPR: False positive rate
hbr: Human brain reference RNA
LC: Human lung cancer
LRT: Likelihood ratio test
MAQC: Microarray quality control project
Med-pgQ2: per-gene Q2 normalization following per-sample median global scaling
MLE: Maximum likelihood estimation
NB: Negative binomial distribution
OC: Human ovarian cancer
PPV: Positive predict value
Q: Full Quantile
Q2: The 50th percentile
Q3: The 75th percentile
QL F-test: Quasi-likelihood F test
qRT-PCR: Quantitative Real-Time polymerase chain reaction
RC: Raw counts
RLE: Relative log estimate
RNA-seq: High-throughput RNA sequencing
ROC: Receiver Operating Characteristic
RPKM: Reads Per Kilobase per Million mapped reads
RSEM: RNA-seq by expectation-maximization
RUV: Remove unwanted technical variation
scRNA-seq: Single cell RNA sequencing
SD: Standard deviation
SRA: Sequenced read archive
TC: Total counts
TCGA: The Cancer Genome Atlas
TMM: Trimmed-mean M values
TNBC: Triple negative breast cancer
TP: True positive
uhr: Universal human reference RNA
UQ: Upper Quartile (Q3)
UQ-pgQ2: per-gene Q2 normalization following per-sample upper-quartile global scaling at 75 percentile
