# Supplementary material for: Choice of library size normalization and statistical methods for differential gene expression analysis in balanced two-group comparisons for RNA-seq studies
Source: BMC Genomics. 2020 Jan 28;21:75. doi: 10.1186/s12864-020-6502-7 (PMC6986029; doi:10.1186/s12864-020-6502-7)
Supplement: Supplementary file 1 — Additional file 1: Figures S1-S4. Illustrated the number of false positive genes identified from intra-group analysis corresponding to Figs. 1, 2, 3 and 4, respectively. [file 12864_2020_6502_MOESM1_ESM.pptx]

## Slide 1
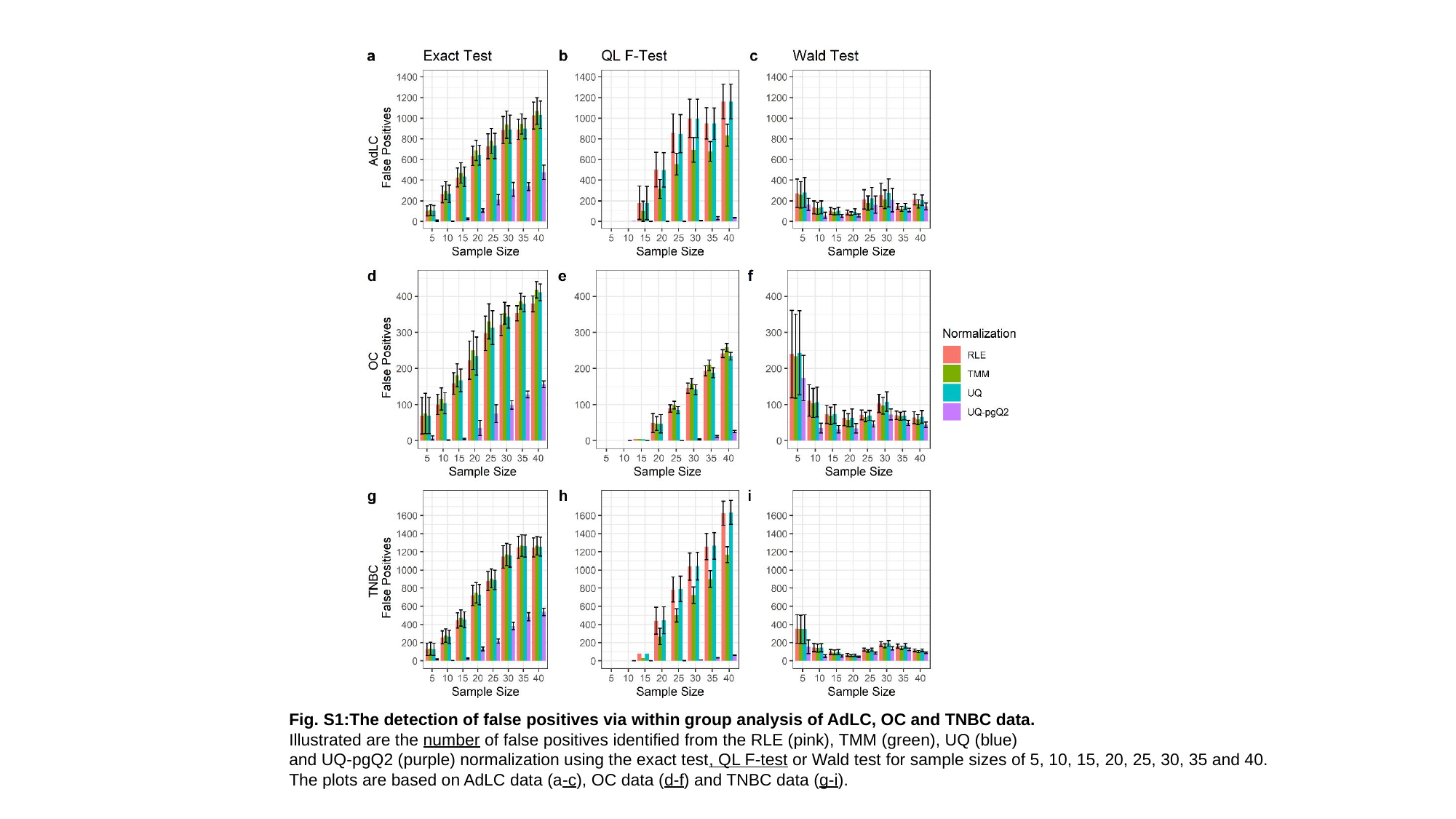

Fig. S1:The detection of false positives via within group analysis of AdLC, OC and TNBC data.
Illustrated are the number of false positives identified from the RLE (pink), TMM (green), UQ (blue)
and UQ-pgQ2 (purple) normalization using the exact test, QL F-test or Wald test for sample sizes of 5, 10, 15, 20, 25, 30, 35 and 40.
The plots are based on AdLC data (a-c), OC data (d-f) and TNBC data (g-i).

## Slide 2
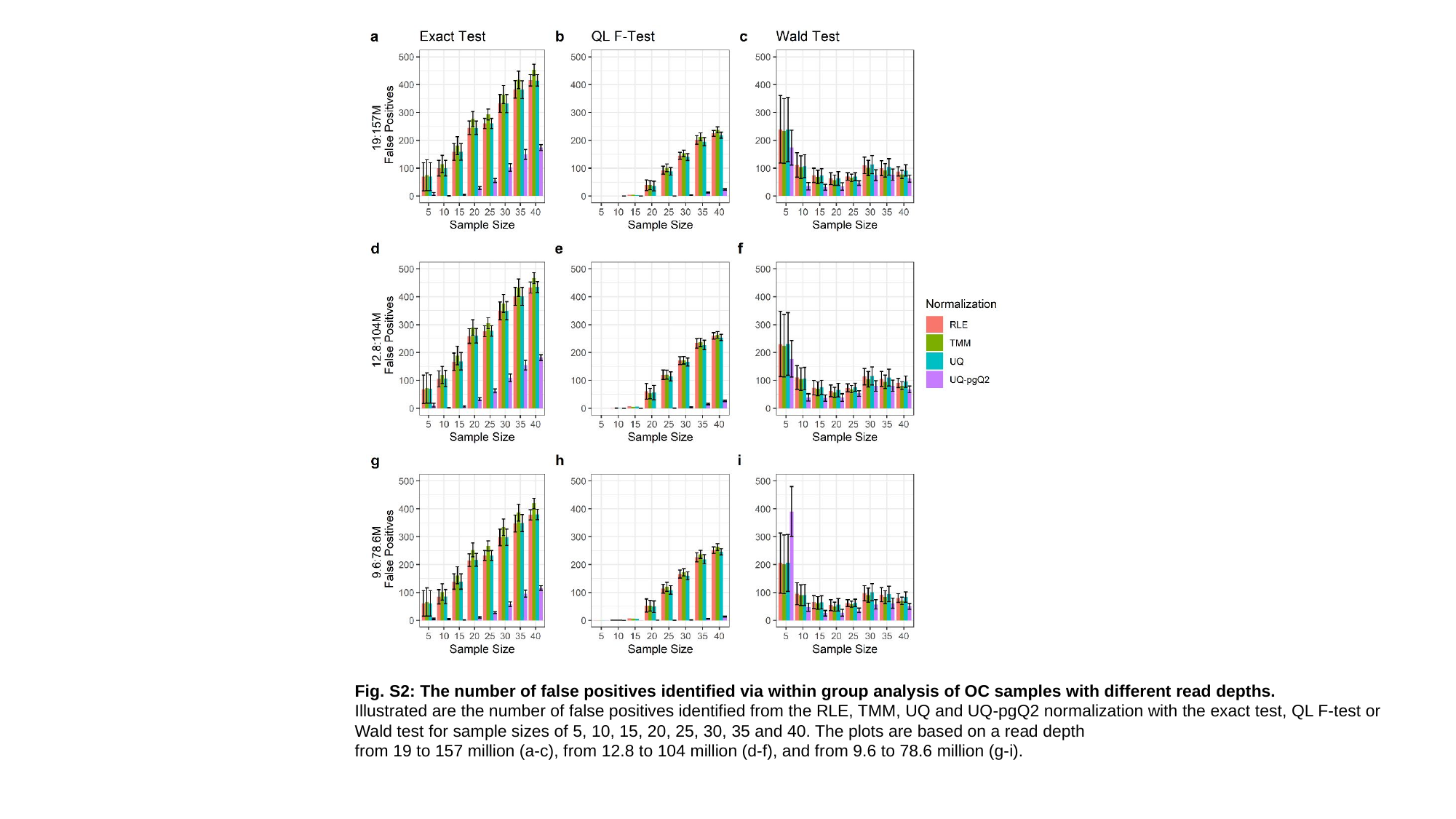

Fig. S2: The number of false positives identified via within group analysis of OC samples with different read depths.
Illustrated are the number of false positives identified from the RLE, TMM, UQ and UQ-pgQ2 normalization with the exact test, QL F-test or
Wald test for sample sizes of 5, 10, 15, 20, 25, 30, 35 and 40. The plots are based on a read depth
from 19 to 157 million (a-c), from 12.8 to 104 million (d-f), and from 9.6 to 78.6 million (g-i).

## Slide 3
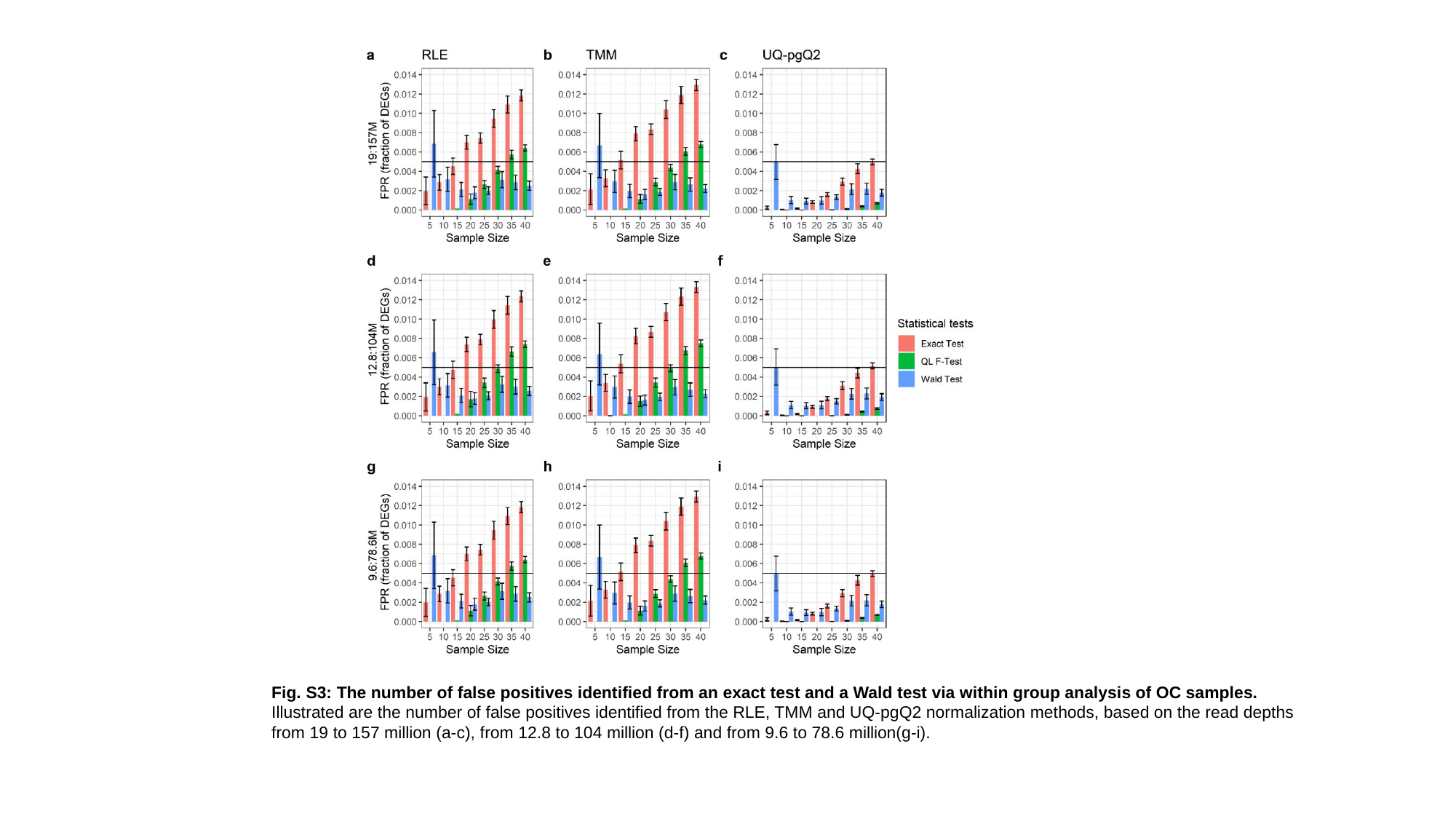

Fig. S3: The number of false positives identified from an exact test and a Wald test via within group analysis of OC samples.
Illustrated are the number of false positives identified from the RLE, TMM and UQ-pgQ2 normalization methods, based on the read depths
from 19 to 157 million (a-c), from 12.8 to 104 million (d-f) and from 9.6 to 78.6 million(g-i).

## Slide 4
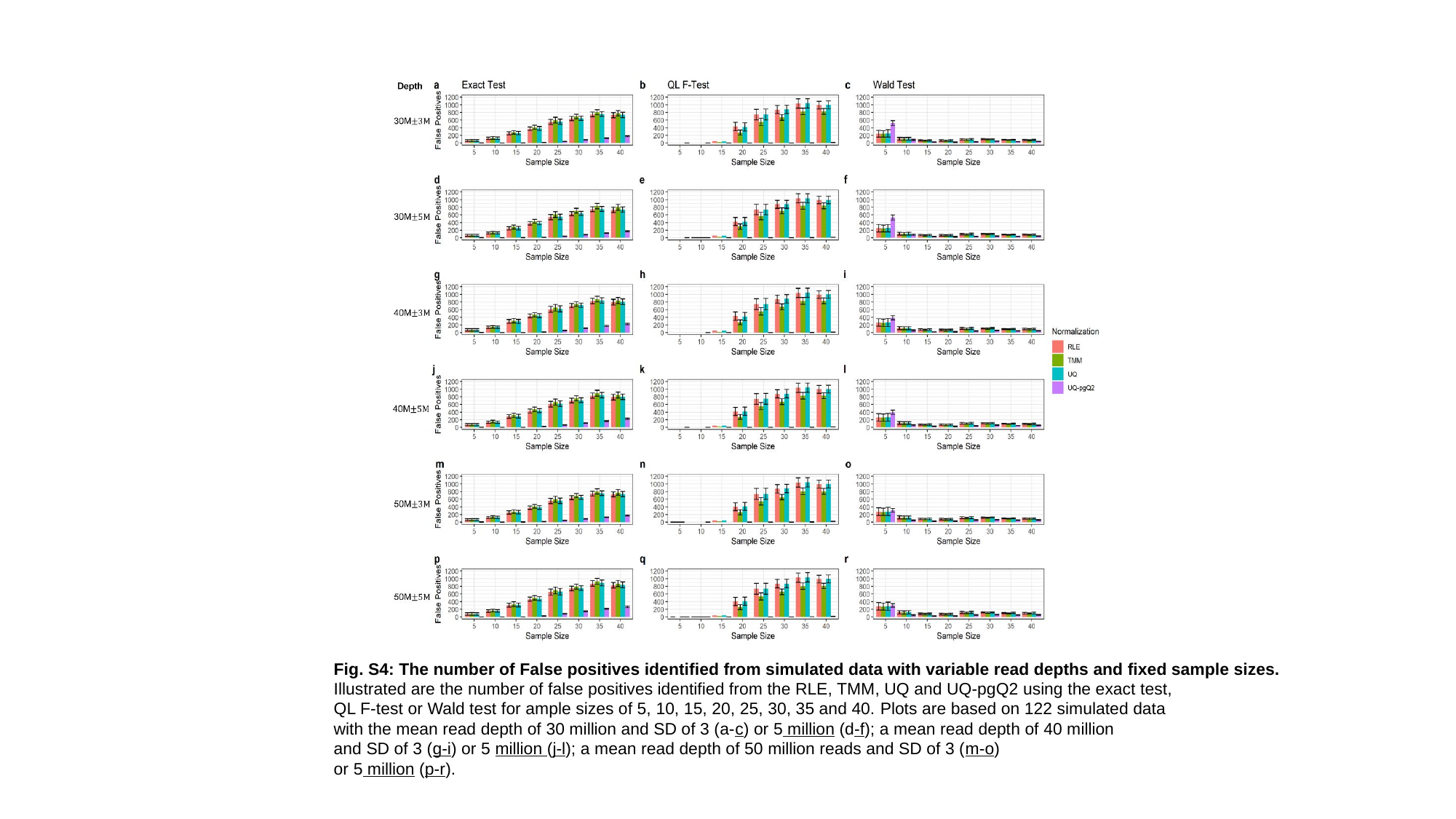

Fig. S4: The number of False positives identified from simulated data with variable read depths and fixed sample sizes.
Illustrated are the number of false positives identified from the RLE, TMM, UQ and UQ-pgQ2 using the exact test,
QL F-test or Wald test for ample sizes of 5, 10, 15, 20, 25, 30, 35 and 40. Plots are based on 122 simulated data
with the mean read depth of 30 million and SD of 3 (a-c) or 5 million (d-f); a mean read depth of 40 million
and SD of 3 (g-i) or 5 million (j-l); a mean read depth of 50 million reads and SD of 3 (m-o)
or 5 million (p-r).
