# Supplementary material for: Choice of library size normalization and statistical methods for differential gene expression analysis in balanced two-group comparisons for RNA-seq studies
Source: BMC Genomics. 2020 Jan 28;21:75. doi: 10.1186/s12864-020-6502-7 (PMC6986029; doi:10.1186/s12864-020-6502-7)
Supplement: Supplementary file 2 — Additional file 2. Contains datasets used for the analysis. This zipped file folder contains MAQC2 and MAQC3 raw read counts, cancer raw data files filtered with zero counts (AdLC, OC and TNBC), and description of these data files named Supplementary Material.docx. [file 12864_2020_6502_MOESM2_ESM.zip › Supplementary Material.docx]

**Supplementary Material**

**Choice of library size normalization and statistical methods for differential gene expression analysis in balanced two-group comparisons for RNA-seq studies**

Xiaohong Li*, Nigel G.F. Cooper, Timothy E. O’Toole, Eric C. Rouchka

**Table S1:**

RNA-seq data were used in the study.

| **File names** | **Genes filtered out with zero > 50% across samples** |
| --- | --- |
| TNBC_122s_RNAseq_rawcounts_filterNA0.5 | Yes |
| OC_rawcounts_filterNA0.5 | Yes |
| AdLC_535s_rawcounts_filterNA0.5 | Yes |
| MAQC2 | No |
| MAQC3 | No |
